# Inferring Human Activity Recognition with Ambient Sound on Wireless Sensor Nodes

**DOI:** 10.3390/s16101586

**Published:** 2016-09-27

**Authors:** Etto L. Salomons, Paul J. M. Havinga, Henk van Leeuwen

**Affiliations:** 1 Ambient Intelligence Group, Saxion University of Applied Science, P.O. Box 70000, 7500 KB Enschede, The Netherlands; leeuwen.henk.van@gmail.com; 2 Pervasive Systems Group, University of Twente, P.O. Box 217, 7500 AE Enschede, The Netherlands; p.j.m.havinga@utwente.nl

**Keywords:** wireless sensor networks, sound, context awareness, feature extraction

## Abstract

A wireless sensor network that consists of nodes with a sound sensor can be used to obtain context awareness in home environments. However, the limited processing power of wireless nodes offers a challenge when extracting features from the signal, and subsequently, classifying the source. Although multiple papers can be found on different methods of sound classification, none of these are aimed at limited hardware or take the efficiency of the algorithms into account. In this paper, we compare and evaluate several classification methods on a real sensor platform using different feature types and classifiers, in order to find an approach that results in a good classifier that can run on limited hardware. To be as realistic as possible, we trained our classifiers using sound waves from many different sources. We conclude that despite the fact that the classifiers are often of low quality due to the highly restricted hardware resources, sufficient performance can be achieved when (1) the window length for our classifiers is increased, and (2) if we apply a two-step approach that uses a refined classification after a global classification has been performed.

## 1. Introduction

Context awareness in home environments can be gained in a number of ways. For humans, some of these methods come naturally. If you close your eyes and listen to your environment, you will probably recognize the voice of other people in the same room, you might be able to infer that your colleague is using his computer because of the clicking sound the keyboard produces, or you could infer that the table is being laid as you hear cutlery and dishes that are taken from a cupboard and put on a table. All of this information can be used by home automation systems to change the lights, heating, or the type of music that is being played. Other uses include home security and facilitating elderly people to live in their own homes longer.

In our research, we investigate the possibilities of wireless sensor networks that use sound to gain context awareness of a home or office environment. Benefits of this approach are that this type of sensing can be used in a non-invasive manner, which is important in terms of acceptability to users. Furthermore, microphones are easy to use and are inexpensive. Other research often uses cameras for this purpose. However, this type of solution is more obtrusive, complicated, and expensive. A downside to the use of wireless sensor nodes is the limited available processing power.

We need to know how to perform human activity recognition on such sensor nodes. In essence, this means that the platform must be able to perform the following three steps: record sound signals, perform feature extraction, and classify the features. As wireless sensor nodes are designed for the purpose of recording sensor data, the first step should not pose a problem. However, the second and third step will depend on the possibilities of executing them on limited hardware.

In a previous article [1], we investigated feature extraction and classification by looking at the work of other researchers that have worked in the area of sound classification. We related the achieved accuracies of their work to the Relative Execution Time (RET) of the feature extraction algorithms. Although this survey gave us insight into the appropriateness of the various algorithms for our approach, some issues remained; mainly related to performance evaluation. One of the challenges we encountered is the difficulty in comparing the recognition success of the various papers. Authors often use different measures to qualify the results, such as Accuracy, Recall, or Equal Error Rate. Additionally, the data sets used for classification and the number of classes to be recognized vary highly.

In this paper, we present a solution for these problems. We evaluate and compare the different algorithms for feature extraction in order to find the best option to be used on wireless sensor nodes. More precisely, we are looking for a set of features that have a low RET for extraction and a high recognition rate when used in classification algorithms. As the comparison of existing research leaves too many questions unanswered, we will perform the classification using different classification schemes ourselves, so that we can be more certain about the applicability of the feature extraction algorithms in varied conditions. For the same reason, we will use sound samples that are collected from different sources. This will deal with the risk of a solution that is tailored for one particular situation.

This article is structured as follows. Section 2 discusses work that is related to this paper. In Section 3, we describe the setup we use for our comparative experiments. Section 4 and Section 5 describe the results and conclusions of the experiments, respectively.

## 2. Related Work

The search for the best set of features and classification algorithms is the subject of ongoing research. A fine example is the recent paper by Barchiesi et al. [2], where different methods of feature extraction and learning algorithms are compared. The different approaches are results from the IEEE AASP Challenge: Detection and Classification of Acoustic Scenes and Events (D-CASE) [3]. Contestants to this challenge were asked to train and test a classifier based on a set of sound samples from 10 different environments, such as an office, a bus, and an outdoor park. The classifiers were compared to classification of the same sound samples by humans. Barchiesi et al. found that the best performing algorithm achieves a mean accuracy that matches the average accuracy achieved by humans. Although their paper provides a good comparison of different approaches, it does not take efficiency of the extraction and classification algorithms into account.

Other work comparing the fitness of different features for sound classification includes the work by El Ayadi et al. [4]. In their survey on emotion recognition, four types of speech features were distinguished: Continuous (Pitch, Energy, Formants), Qualitative (Voice quality, harsh, tense, breathy), Spectral (Linear Predictive Coding, Mel-Frequency Cepstral Coefficients, Log-Frequency Power Coefficients), and TEO-based. The latter category is based on the Teager-Energy-Operator, which was developed using the notion that hearing is in fact a process that detects energy in a sound wave. The TEO features are specifically aimed at detecting stress in speech. With respect to the types of classifiers that are being used (e.g., Hidden Markov Models, k-Nearest Neighbors classifiers), El Ayadi et al. note that there is no notable advantage of using one type of classifier over the others. Again, their paper does not take efficiency of feature extraction into account.

Besides the method of using a fixed set up of sensors in a room, other approaches also appear in the literature. Shoaib et al. [5] present a survey of the methods that are currently in use for on-line activity recognition using mobile phones. A good overview of recent research on multi-occupant activity recognition is presented by Benmansour et al. [6].

## 3. Experiment Set-up

### 3.1. Dataset

The aim of our system is to gain context awareness in home environments using sound. We indicate three categories that are of relevance for this purpose. First, we classify sound as either *voice*, *music*, or *environment*, which enables a more focused classification of the concerned sound as a second step. We denote this first category as *Global*. The three classes of this category can each serve as a category of its own. Although music can give a global indication of the activities in a room, it will be hard to directly link an activity to a certain music style. We therefore chose not to dig deeper into this category. The other classes—*Voice* and *Environment*—will be elaborated further. For the *Voice* category, we are only interested in the person type we hear, rather than in identifying particular persons. Moreover, detecting particular persons requires higher performance hardware than is present on realistic wireless nodes. In the *Environment* category, we restrict ourselves to indoor sound events.

A context-aware application should be able to perform its duties in very different environments, as we will encounter in homes and other buildings. This is why we chose not to record our own sounds, but rather to use sound files that were recorded by different people, in order to mimic this variety. As we want our results to be verifiable by other researchers, we used files that are publicly available. The site Freesound [7,8] proved to be a valuable and suitable source. The site contains a high number of sound samples recorded by people with diverse interests. The sound files are shared under Creative Commons licenses and are used for research as well as artistic purposes.

For the classification of the gender of voices, we use audio books we downloaded from LibriVox [9]. LibriVox audio books are public domain recordings, created by volunteers. It contains over 9000 projects in multiple languages.

For the classification of music, we selected a number of songs from various artists and composers.

As a consequence of using sound files form many different sources, we expect that it will be hard to achieve high recognition rates. That is not problematic for this research, as we are mainly interested in the differences between the classification results using the various approaches.

The size of the used datasets is shown in Table 1. We select at least five different sources for each sound type. For some classes, the total length of the sound files is considerably longer than the length of the other classes. This is due to the fact that files of some sound source types are more easily obtained than others. Within each class, however, the sound files are of comparable size. As the classifiers we train create a per-class model, this difference in size does not influence our results. We find that the total length of these sound waves is adequate for the training of our classifiers.

### 3.2. Training and Classification

**Figure 1:**
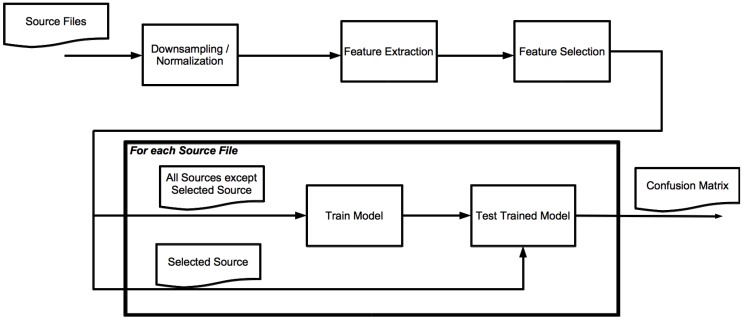
Classification process.

The process of training and classification is depicted in Figure 1. We first ensure that all sound files are comparable in sound volume and quality. As we want to be able to deploy our algorithms on wireless sensor nodes, we have to take into consideration the limits of this type of hardware. As described by Ganchev et al. [10], the sampling frequency of 8 kHz is common for all telephone-driven services, and is thus the default for real-world speaker recognition corpora. As this sampling frequency does not exceed the capabilities of contemporary hardware for wireless sensor networks, we chose to perform our experiments using this frequency for our files. Because of this, all sound samples in our tests are down-sampled to this frequency. Furthermore, we want the amplitude of our sound sources to be comparable to each other. For this reason, we applied amplitude normalization before we proceeded to the next step. In a real environment, loudness provides extra information on the sound producer, such as distance from the microphone. As we work with samples that are recorded in different set-ups, it is best to eliminate these differences.

We chose not to filter silence from the sound signals before processing. The reason for this is that some sounds contain specific and characteristic patterns of silence and non-silence, which can be an important feature for the classification. For example, speech generally contains pauses, whereas music is generally without silence. Some classification algorithms, such as Hidden Markov Models (which we will describe in Section 3.4.2) are particularly suitable for the detection of these patterns. In order to gain insight into the different performance of our classification algorithms, we use the same parts of the original signal for the training of each classifier.

There is a downside to this, however, as silence by itself cannot be used to distinguish between activities. It is therefore to be expected that some classifiers will benefit from the removal of silence before classification, and doing so is not complicated. However, as we are mainly interested in gaining insight into the feasibility of classification on wireless nodes, we will not perform this step for now.

Our next step is to extract features from the sound files. The following types of features are extracted:

- Time Domain (TD) features (Zero Crossing Rate, Short Time Energy): low-cost features that do not need transformation to the frequency domain. This type of feature has the computational order of complexity O(n).
- Frequency Domain (FD) features (Fundamental Frequency, Bandwidth, Spectral Centroid, Spectral Roll-off): require Fast Fourier Transforms (FFT) to be performed; this offers more load on the processors, but is necessary for more advanced recognition strategies. Press et al. [11] argue that the FFT can be calculated in O(nlogn) complexity. All Frequency Domain functions require an additional O(n) step after calculation of the frequency spectrum, so they each have the same order of magnitude as the calculation of FFT.
- Mel Frequency Cepstral Coefficients (MFCC): computation-heavy, but widely used for all kinds of recognition experiments with respect to sound. MFCCs can be regarded as the current de facto standard for sound, speaker, and speech recognition. MFCC use the FFT transform as one of the main steps. Other steps that are performed in the algorithm have lower complexity than the calculation of the FFT. Therefore, the complexity of this algorithm can be expressed as O(nlogn).

More details about the algorithms can be found in [1] and Appendix B.

All features are extracted using 32 ms frames, which is in the range commonly used for feature extraction. Frames of this size can be regarded to be statistically stationary. For shorter frames, the number of samples would be too small to get a reliable spectral estimate.

In order to train a system that is as independent of individual sources as possible, we train and test in a one versus all strategy. For each source file, we train a classifier using all remaining sources. The classifier is then tested using the selected source.

### 3.3. Window Voting

The short 32 ms frames we use for feature extraction work well for a number of features. If we only consider frames that are that short, we have a risk of losing information, as we do not look at adjacent frames. That is why we use windows of 0.25, 0.5, 1, and 2 s. Based on the classification results of Figure 1, we count how many frames within a window are classified as a certain class. The class with the highest count wins. We call this process *Window Voting.*

This approach could result in a recognition rate that is higher or lower than the actual frame-by-frame recognition of the classifier. That is why we calculate the confidence for each window, which is defined as the fraction of the class count divided by the total number of frames in a window.

### 3.4. Classification Algorithms

There are multiple algorithms for the classification of feature vectors. In this paper, we describe two approaches: Support Vector Machines (SVM, Section 3.4.1) and a combination of *k*-means and Hidden Markov Models (KHMM, Section 3.4.2). Both approaches demand a higher processing power than can be offered by most wireless sensor nodes during the training phase. However, as these approaches are highly different, using both algorithms will give insight into the suitability of each feature combination for the purpose of classifying environment sounds. If both methods lead to the same conclusion, we can be more certain that this conclusion holds under all conditions. Our next step will then be to find a proper classification algorithm that can be deployed on resource-constrained devices.

#### 3.4.1. Support Vector Machines

The use of Support Vector Machines (SVM) is well-established for classification problems. Although SVMs are binary classifiers, there are methods of extending this classifier to multiple classes. For our experiments, we use LIBSVM [12], which has a one-versus-one approach for the classification of multiple classes.

The computational complexity of the training and classification process of a SVM depends on the dimensionality of the vectors *d* and the number of vectors used for training *n*. According to Chapelle [13], the training complexity can be expressed as $O(max\left(n,d\right)\cdot min{\left(n,d\right)}^{2})$ for non-linear kernels. As the number of vectors we use for training is 1000 or higher, this means that the training of the nodes can best be performed off-line on a desktop computer. At test time, the complexity is linear on the number of support vectors.

#### 3.4.2. *K*-Means-Hidden Markov Model

**Figure 2:**
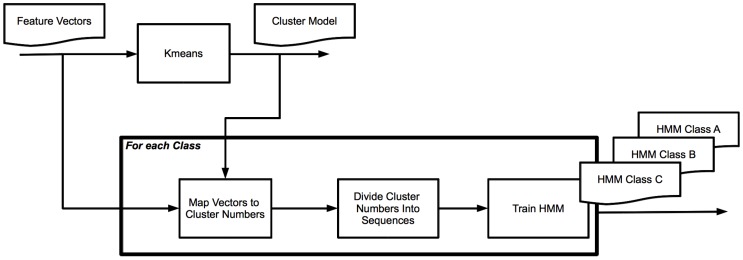
*k*-means and Hidden Markov Model (KHMM) training.

The *k*-means-Hidden Markov Model (KHMM) approach is inspired by Yi Zhan [14]. Rather than using the features of individual frames for classification, this approach takes the development over time of the sound signal into account. The use of HMMs allows us to determine the likeliness of a sequence of observations (in our case: sequence of feature vectors) given a model that is created using observation sequences that belong to a certain type. In order to be able to use HMMs, we therefore need to map the possible feature vectors to a limited set of observations. The following steps are taken for training the KHMM (see also Figure 2):

1. Using the feature vectors from all sound types, apply *k*-means to find clusters of vectors. Each cluster represents one type of observation. Clusters are numbered from *0* to *k*−1.
2. For each sound type, map the feature vectors to cluster numbers based on the model created in the previous step.
3. Subdivide the series of cluster numbers into sequences of a length that corresponds with the chosen window size and the number of frames per second.
4. For each sound file, create observation sequences that are based on the clustering method of the previous step. Train an HMM for this type.

For the classification of new samples, we use the cluster model from the first step to map the feature vectors to observation numbers. As in the training phase, this list of observations is subdivided into sequences of a length that corresponds with the chosen window size.

For each sound file that we want to classify, we create observation sequences and try to match the sequence with each HMM. The sound type of the HMM that has the highest probability is chosen as the result of our classification.

There are a number of parameters that can be chosen: the number of clusters (value of *k*), the number of hidden states of the HMM, and the length of the observation sequences we use for training and testing. We start using Zhan’s advice by using 15 clusters, 7 hidden states, and observation sequences that correspond to 1 second of sound and use a number of variations of the parameters for comparison.

Rodriguez and Torrez [15] describe the computational complexity of finding the correct parameters for a Hidden Markov Model as $O(3N^{2}T+NT+NTRC)$, where *N* is the number of hidden states, *T* is the length of the observation sequences, *R* is the number of observation sequences, and *C* is the number of symbols in the discrete output distributions. Calculation of the probability of a model, given an observation sequence, can be expressed as $O(N^{2}T)$.

The complexity of the k-means clustering algorithm is $O(n^{dk+1}\log n)$ for the training phase, according to Inaba et al. [16], where *n* is the number of samples to be clustered, *d* is the dimension of the vectors, and *k* is the number of clusters. Classification of a given vector is linear with *k*.

Given these complexities, training this model will only be possible on a more powerful platform than a wireless sensor node. Classification on a resource-constrained node should not be a problem given the orders of complexity.

### 3.5. Classification Score

The results of the classifications can be presented as a confusion matrix. Based on this matrix, we quantify the success rate of the classification by using the $F_{1}$ score, which is defined as $F_{1}\;=\;\frac{2.\mathrm{precision}.\mathrm{sensitivity}}{\mathrm{precision}+\mathrm{sensitivity}}$. The success rate depends on two quantities: *precision*, or the class agreement of the data labels with the positive labels given by the classifier; and *sensitivity* (also known as *recall*): the effectiveness of a classifier to identify positive labels. Mathematically, these are expressed as $\mathrm{precision}=\frac{TP}{TP+FP}$ and $\mathrm{sensitivity}=\frac{TP}{TP+FN}$, where *TP = True Positives*, *FP = False Positives* and *FN = False Negatives*. We have to use these quantities with caution, however. If the number of samples of one class is much higher than of other classes, this can have an unwanted negative effect on the precision of these classes. We deal with this problem by putting the fractions $\frac{nr(C_{A,X})}{nr(X)}$ in the confusion matrix instead of absolute values, where $nr(C_{A,X})$ is the number of samples from class *X* that are labelled *A*, and nr(X) is the total number of samples from class *X*.

### 3.6. Testing Platform

The computational complexity of training the classifiers is too high for wireless sensor nodes (see Section 3.4). Fortunately, this step has to be taken only once, and can therefore be done off-line on a desktop computer. The main purpose of our system—classification of environment sounds—will be performed on the wireless sensor nodes once the classifiers have been created and uploaded.

To test whether the classification algorithm can actually be deployed on a wireless sensor network, we ran performance tests and evaluated the results using the Jennic JN5148 module from NXP. This module is a good example of a wireless network device. It features a low power consumption, yet has a relatively high-performance microprocessor unit combined with an IEEE802.15.4 compliant transceiver. The microprocessor of this module consists of a 32-bit RISC CPU and has 128 kB available for program code and data. It has a 12-bit Analog-to-Digital Converter (ADC) that is capable of sampling sound at 25 kHz. For our purposes, however, 8 kHz is enough. This frequency leaves us with phone-quality audio signal, which covers our needs. Although higher frequencies will result in better classifications, the downside is that both memory usage and processing time will increase.

In prior experiments, we used the Jennic JN5148 platform to determine the RET for feature extraction algorithms [1].

## 4. Results

Before comparing classification algorithms, we first selected which features to use (Section 4.1). The actual results of the experiments are described in Section 4.2, Section 4.3 and Section 4.4. In Section 4.5, we describe the deployment of the classifier on wireless sensor nodes.

### 4.1. Feature Selection

In previous work [1], we calculated the Relative Execution Time (RET) for frequently-used features for sound recognition (see Table 2). The RET is a measure of the effort necessary for a certain type of calculation. After initial tests, some features were discarded for subsequent experiments, for the following reasons:

- Haar-like features, introduced by Jun Nishimura [17] and Yi Zhan [14], are described to be highly discriminative. These features are interesting, as only limited processing power is needed for the calculation. We followed Zhan’s proposed method on our dataset. We trained KHMM models using all combinations of five filters ranging in length from 2 to 20. The results were disappointing, however; for most categories, the classification results were similar or even lower than the results obtained by using other time-domain features. The only exception was the classifier that was trained to distinguish voice from environment sounds. The classifier for this category had a slightly better performance when haar-like features were used instead of other time-domain features.
- Linear Predictive Coefficients. These features were often used prior to the introduction of Mel Frequency Cepstral Coefficients (MFCCs). They are slightly less computationally intense than MFCCs, but are less discriminative.
- Long-Term Features (Jitter, Shimmer). The RET of these features is comparable to TD and FD features. During our experiments, we found that these features did not contribute positively to a better classification result.

Three feature types remain of interest: Time Domain features (TD), Frequency Domain features (FD), and Mel Frequency Cepstral Coefficient features (MFCC). We experiment with each of these features types by themselves and in the combinations (TD, FD) and (TD, MFCC). As the MFCC is dependent on frequency analysis, we see no added value in investigating the combination (FD, MFCC).

### 4.2. SVM Classification Results

For each of the categories mentioned in Section 3.1, we performed the classifications with some variations:

- For the category *Global*, we considered the classification of *<voice, music, environment>* and *<voice, environment>*.
- For the category *Gender*, we considered the classification of *<child, female, male>* and *<female, male>*.
- For the category *Environment*, we classified using all available classes: *<chair, dishes, hand dryer, rain, shaving, shower, toilet, toothbrush, typing, urinate, vacuum, walking>*. We also performed classification using subsets of sounds that are likely to occur in a specific location. We distinguish office sounds *<conversation, typing, vacuum, walking>*, bathroom sounds *<hand dryer, shaving, shower, toilet, toothbrush, urinate>*, and kitchen sounds *<conversation, cooking, cutlery, dishes, pan>.* In addition to the available environment classes, we included *conversation* sounds to the office and kitchen environment, as these are likely to occur in this type of environment.

Table 3 shows the results of the classification experiments that use a SVM to train the classifier. The feature columns are ordered from low to high RET. The RETs from Table 2 are shown in the table header. In order to provide more insight into the classification results, we included the confusion matrices of the classification using the MFCC features in Appendix A (Figure A1, Figure A2, Figure A3, Figure A4, Figure A5, Figure A6, Figure A7, Figure A8, Figure A9, Figure A10, Figure A11, Figure A12, Figure A13, Figure A14, Figure A15 and Figure A16).

We observe that the classifications of categories with higher numbers of classes have reduced success rates compared to classifications with a lower number of classes. This is not unexpected, as it is generally easier to distinguish between a limited number of classes.

As was to be expected, the experiments that included MFCC scored higher than experiments with other features. Although we expected to see better results for the combination of MFCC and time domain features, this was not the case in our experiments. On the contrary, for most categories, the use of only MFCC features produced better results than using it in combination with time domain features, although the difference in $F_{1}$ scores was small (~0.01) in most cases.

For the cases in which only time domain and frequency domain features are used, the combination of both features generally delivers better results than each of these two types of features apart. A notable exception is the *kitchen* class, which has a particularly low score for the experiments that include frequency domain features.

Of all categories, the experiments involving global category type sounds had the highest recognition rates. The difference between using MFCCs and the TD + FD combination was 0.1 in favor of MFCCs.

**Figure 3:**
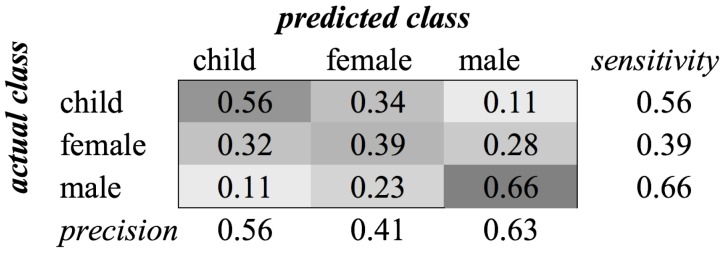
Classification of category Gender using SVM on time domain (TD) + frequency domain (FD) features.

For the gender category, the recognition rates using these features were almost equal. The reason for this small difference is that the base frequency of a voice contains a strong indication of the gender of a person. The scores of classification with and without the child class are approximately 0.15, which is a huge difference. Figure 3 shows the confusion matrix of this classification for the TD + FD combination. The matrix for MFCC features is similar. We see that the classifier had difficulties distinguishing female voices from both male and child voices. The best performance was achieved for classifying male voices. We assume that the lower frequencies of the male voice contributed to this distinction, whereas the higher frequencies of female voices and children’s voices are closer to each other. Without the child class, experiments resulted in an average $F_{1}$-score of 0.72 when MFCCs are used.

**Figure 4:**
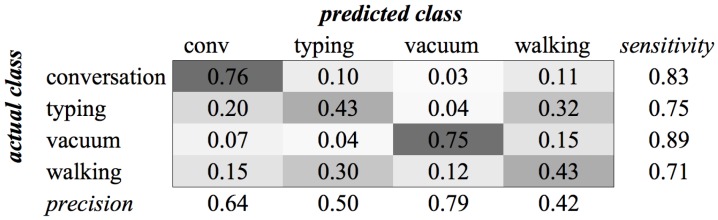
Classification of Category Office using SVM on MFCC features.

**Figure 5:**
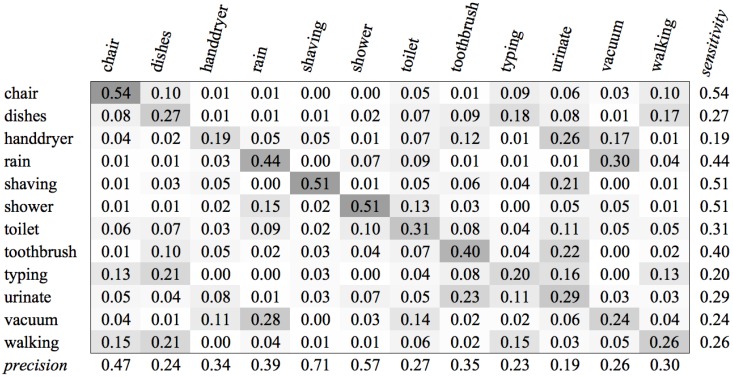
SVM Confusion Matrix of Category Environment for MFCC feature type.

The experiments for the various environment classes have very low scores. The highest score, 0.59, was achieved for the classification of sounds of the subcategory Office (see Figure 4). We see in the confusion matrix that the sound sources that produce a more constant noise (like conversation and vacuum cleaners) result in higher recognition rates than the sounds that have a more staccato type of signal. When all environment classes are the subject of our recognition experiment, we are only able to achieve a score of 0.35 (see Figure 5).

### 4.3. Window Voting Results

**Figure 6:**
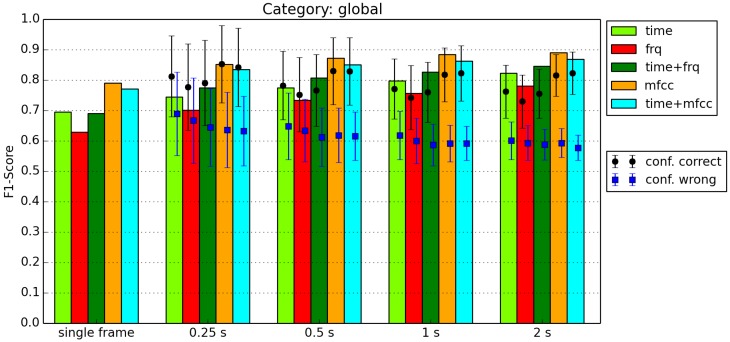
Classification of category Global.

An example of the results of Window Voting (see Section 3.3) is shown in Figure 6. For each window size and feature combination, the voting result and the confidence of the correctly classified and of the incorrectly classified windows are shown. The black dots and blue squares show the average confidence, and the connected bars indicate the size of the standard deviation. Figure 6 shows a typical result for our classifications. The $F_{1}$ scores gradually increase for longer windows, and the confidences gradually decrease. In this example, the confidences of the incorrect classifications are about 1 standard deviation lower than the classifications of the correctly classified windows. For other categories, however, these values are closer to each other. In some cases, the average confidence for the incorrectly classified windows is actually a little bit higher.

We had hoped for the average confidence of correctly and incorrectly classified windows would be further apart. For most categories, however, the averages of the confidences are generally less than one standard deviation apart. The only case in which the confidences are further apart was observed for the global category when using windows of 0.5 s or longer and classifying using MFCC features. In this particular case, we could leverage the confidence level to decide whether or not to accept a classification result. Based on Figure 6, if we use 2 s windows, for example, we accept a classification based on MFCCs if the confidence of the window voting is higher than 0.75 and reject the classification if the confidence is lower than 0.65. All confidences in between result in an “uncertain” classification.

### 4.4. KHMM Results

Table 4 shows the results from the classification using the KHMM approach for a window of 0.25 s. For comparison, we also included in this table the results from the SVM classifier when using window voting on a 0.25 s window.

We performed the KHMM approach several times, using different values for the number of clusters and the number of hidden states. We followed Zhan’s suggestion to use 15 clusters and 7 hidden states first, and then performed experiments with all possible combinations of cluster numbers 15, 10, and 5, and number of hidden states 10 and 5. Although some configurations resulted in small improvements for certain categories, there was not one configuration that was beneficial for all categories. For the overview of Table 4, we used the proposed 15 clusters–7 states configuration.

In some cases, the classifier was not able to create a model for one or more classes. To create a valid HMM, the observation sequences used to train need to contain each observation at least once. Apparently, the feature vectors of some classes only occupy a limited space compared to other classes. These cases are indicated with a dash in the table.

Beforehand, we expected the KHMM approach to be better than using SVM. Our hypothesis was that this approach would benefit from knowledge of time characteristics in the signal, for example from the frequency of silence within a sound signal. In our experiments, we found no evidence for this hypothesis. In fact, the opposite was true: the KHMM approach resulted in better results for only three out of 32 experiments. In the experiments with kitchen sounds that use frequency features for classification, the KHMM approach was not able to create a classification model.

Comparing the SVM results in Table 3 and Table 4, we see the benefit of using majority voting. On average, the results are 0.045 higher than for the classification of single frames. The classification of global sounds and gender benefit more from window voting than the classification of environment sounds.

### 4.5. Performance on Wireless Node

**Figure 7:**
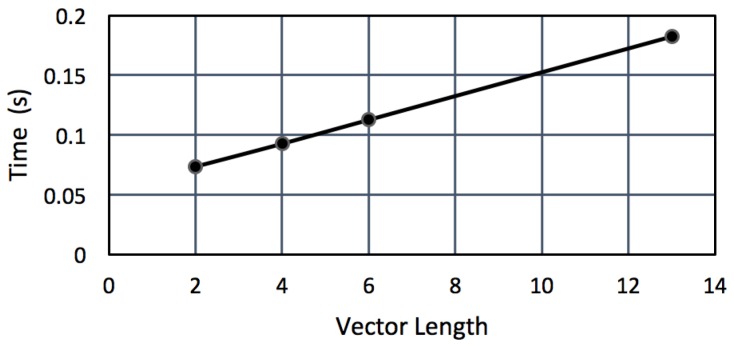
SVM classification times on a JN5148 node with 100 vectors.

To test whether it is actually possible to run the classifier on a wireless node, we uploaded trained classifiers to the Jennic JN5148. Figure 7 shows the average time to classify a vector per 100 feature vectors in the SVM. On average, the trained SVMs in our experiments consisted of 400–600 vectors. The presence of fewer vectors had a positive effect on the classification time, but a negative effect on the classification results.

As we want to know how these timing results relate to the feature extraction times, we assume a SVM that consists of 500 feature vectors. Table 5 shows the RET of feature extraction versus classification using a SVM of this size. We see that the classification time for TD and FD features is approximately the same as the feature extraction time of FD features; the classification time of an MFCC feature vector is about half the time of calculating the vector.

If we consider the total time necessary to calculate one feature vector and to classify it on a Jennic JN5148 node, we can conclude that it is possible to process three frames per second when using TD features, three frames per two seconds if FD features are used, and we can process one frame per three seconds if we use MFCC features.

## 5. Discussion

We compared the classification results with the purpose of finding the features with a low RET and a high classification score. For the global classification, the low-RET features achieve a score that is 0.1 below the high-RET scores. Using slightly longer windows, even as small as 0.25 s, we obtain recognition results with an $F_{1}$ score of 0.8. For this category, we could even use the fastest feature type at a minor loss of accuracy. This knowledge helps to create a two-step recognition approach. As a first quick step, sound is classified as either environment sound or human voice. In the second stage, a more to-the-point classification of the actual sound type can then be performed.

One of the considerations is the length of the window that is used for classification. As we saw in our experiments, using longer windows results in better recognition results. Whether this notion can be used in an actual sound-based context-aware application depends for a large part on the nature of each sound type. It would be helpful to perform measurements in real homes and offices to gain more insight into the duration of particular sounds and whether and how often sound events take place simultaneously or in isolation. If the duration of sound events is longer—which can be the case for music and dialogues between people—there are less concerns regarding extending windows to one second. In other cases that are more staccato in nature (such as sounds of cutlery), longer windows will be less beneficial to achieving a high classification accuracy.

Regardless of the features we choose for our sound recognition task on wireless nodes, we will have to deal with the fact that we have a modest performance for some types of sound. We see that classification attempts with a high number of classes result in lower success rates. In these cases, we notice, however, that even though the $F_{1}$ score of some experiments are very low, the classifiers still manage to achieve a score that is well above random guessing level. As an example: there are six bathroom classes. Random guessing would result in an $F_{1}$ score of 0.17, whereas in our experiments, even the time domain features help to achieve scores of 0.3.

A possible solution to the problem of low scores that is worth investigating is to take the next step towards activity recognition. After classification of the individual frames, we could use the series of recognized classes to train a Hidden Markov Model for the activities that are likely to occur in the concerning environment. Individual misclassifications will then have less impact on the result, as these misclassifications are likely to occur both in training and testing.

From our classifications of environment sounds, it is clear that there is a huge benefit to using the context of the sensor. If a kitchen sensor is trained to classify any type of environment sound, its success rate will be far below the recognition rate of a sensor that is trained to distinguish between kitchen sounds only.

El Ayadi et al. [4] noted in their experiments on emotion classification that the classification results of the various algorithms are of an equivalent level. This is supported by our research results in comparing results from SVM and KHMM. This means we can focus on using the classifier that has the lowest processor footprint for application in wireless sensor nodes, as the heavyweight algorithms do not necessarily produce better results.

In our experiments, we performed the recognition on sound waves that have an 8 kHz sampling rate. This enables us to work with sound frequencies up to 4 kHz. In further research, it will be interesting to see what happens if the sampling rate is reduced to 4 kHz or below. This will be beneficial to the number of frames the wireless node is able to process per second. We do not know, however, what the effect will be on the recognition rate of our classifiers. If there is only a marginal decrease in performance, this will be a better choice for the recording of sound.

## Figures and Tables

**Table 1:** Dataset sizes.

Category	Class	No. Sources	Time	No. Frames
Voice	child	5	00:06:51	12857
	female	12	02:45:12	309749
	male	8	01:56:13	217893
Environment	chair	6	00:02:29	4653
	conversation	6	00:05:27	10208
	cooking	11	00:06:12	11629
	cutlery	8	00:01:33	2903
	dishes	7	00:11:15	51999
	hair-dryer	7	00:05:24	10131
	pan	5	00:01:42	3191
	rain	5	00:05:56	11121
	shaving	6	00:03:08	5875
	shower	6	00:06:04	11362
	toilet	7	00:03:53	7269
	toothbrush	5	00:02:18	4306
	typing	6	00:03:04	5755
	urinate	6	00:02:58	5563
	vacuum	6	00:07:06	13308
	walking	7	00:02:50	5307
Music	music	8	00:03:28	6514

**Table 2:** Relative execution time (RET).

Feature	Abbreviation	RET
Time domain features	TD	1
Haar-like features	Haar-like	3.5
Frequency domain features	FD	15
Long-term features	long	15
Linear Prediction Cepstral Coefficients features	LPCC	30
Mel Frequency Cepstral Coefficients features	MFCC	78

**Table 3:** Support vector machine (SVM) classification Results, RET in brackets for each feature.

Category	No. Classes	TD (1)	FD (15)	TD + FD	MFCC	TD+MFCC
				(16)	(78)	(79)
Global	3	0.70	0.63	0.69	0.79	0.77
Env-Voice	2	0.76	0.76	0.77	0.86	0.86
Gender	3	0.38	0.56	0.57	0.58	0.58
Gender (no child)	2	0.59	0.70	0.73	0.72	0.71
Environment	12	0.17	0.25	0.27	0.35	0.34
Bathroom	6	0.29	0.33	0.35	0.48	0.43
Kitchen	5	0.42	0.07	0.06	0.47	0.46
Office	4	0.38	0.45	0.44	0.59	0.57

**Table 4:** Classification Results for window size 0.25 s. RET in brackets for each feature.

Category	No.	TD (1)			FD (15)			TD + FD (16)			MFCC (78)		
	Classes	*svm*	*khmm*	*diff*	*svm*	*khmm*	*diff*	*svm*	*khmm*	*diff*	*svm*	*khmm*	*diff*
Global	3	0.74	0.75	***−0.01***	0.70	0.68	***0.02***	0.78	0.70	***0.08***	0.85	0.84	***0.01***
Env-voice	2	0.80	0.81	***−0.01***	0.83	0.80	***0.02***	0.82	0.80	***0.02***	0.92	0.89	***0.03***
Gender	3	0.39	0.38	***0.01***	0.60	0.51	***0.09***	0.63	0.46	***0.17***	0.66	0.64	***0.02***
Gender (no child)	2	0.64	0.63	***0.01***	0.77	0.62	***0.15***	0.81	0.60	***0.20***	0.82	0.79	***0.03***
Environment	12	0.17	0.12	***0.05***	0.28	0.15	***0.13***	0.30	0.10	***0.19***	0.41	0.33	***0.09***
Bathroom	6	0.30	0.16	***0.15***	0.37	0.25	***0.12***	0.40	0.28	***0.12***	0.55	0.55	***0.00***
Kitchen	5	0.45	0.38	***0.07***	0.07	-	***-***	0.06	-	***-***	0.53	0.51	***0.02***
Office	4	0.39	0.38	***0.02***	0.48	0.48	***0.00***	0.46	0.49	***−0.04***	0.63	0.61	***0.02***

**Table 5:** Relative execution time of feature extraction vs. SVM classification.

Feature	Feature Extraction	SVM Classification
Time Domain features	1	15
Frequency Domain features	15	19
MFCC features	78	38

**Figure A1:**
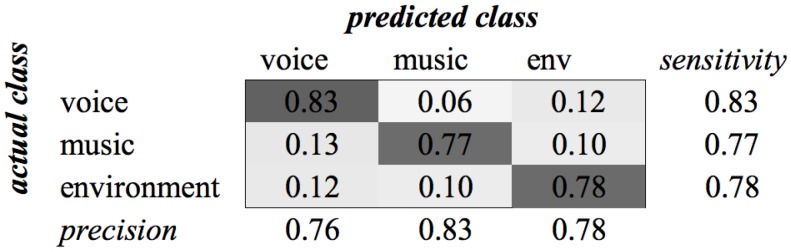
SVM confusion matrix of category Global for MFCC feature type.

**Figure A2:**
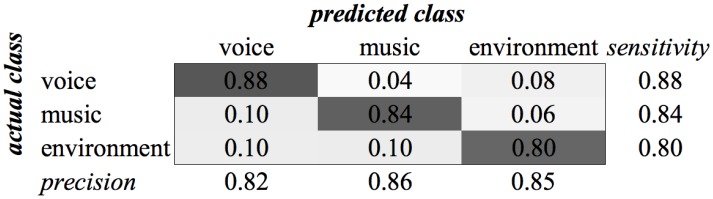
KHMM confusion matrix of category Global for MFCC feature type.

**Figure A3:**
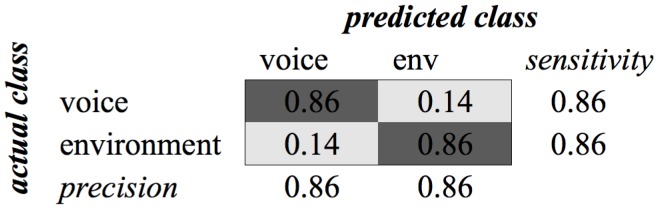
SVM confusion matrix of Environment/Voice classification for MFCC feature type.

**Figure A4:**
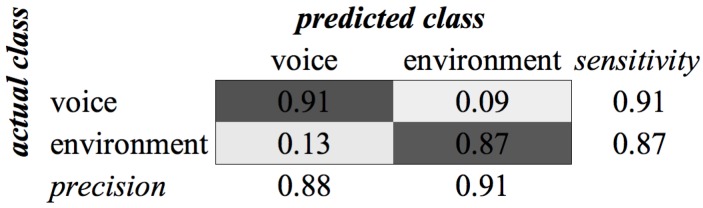
KHMM confusion matrix of Environment/Voice classification for MFCC feature type.

**Figure A5:**
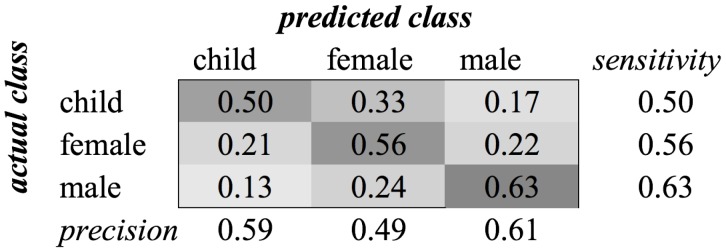
SVM confusion matrix of category Gender for MFCC feature type.

**Figure A6:**
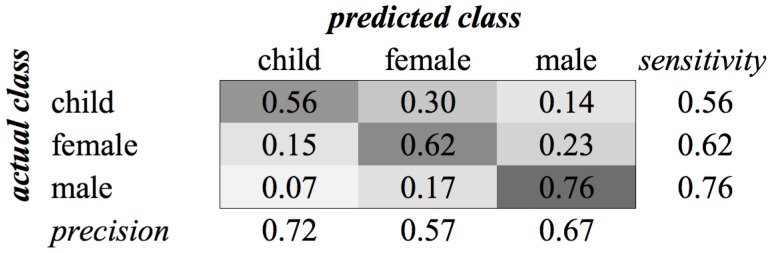
KHMM confusion matrix of category Gender for MFCC feature type.

**Figure A7:**
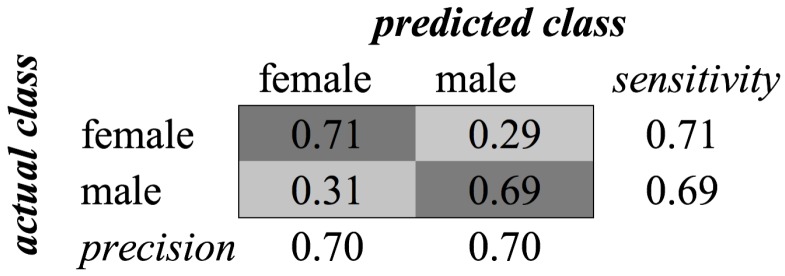
SVM confusion matrix of category Gender (without child class) for MFCC feature type.

**Figure A8:**
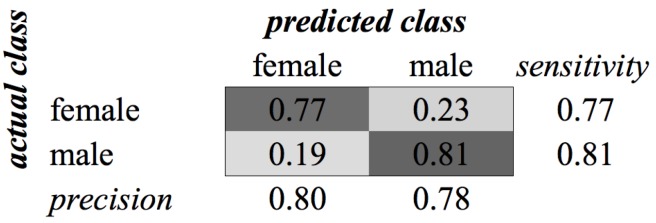
KHMM confusion matrix of category Gender (without child class) for MFCC feature type.

**Figure A9:**
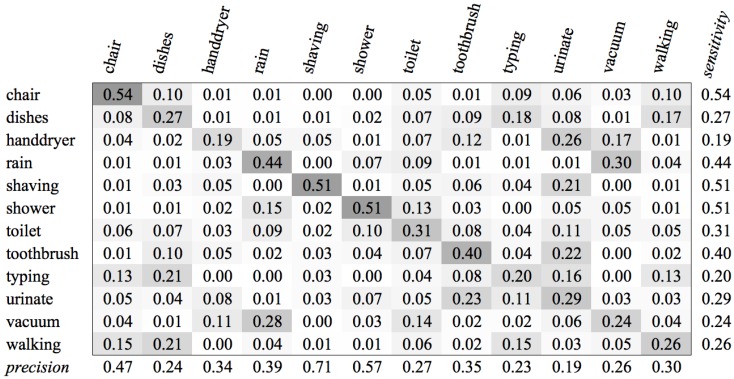
SVM confusion matrix of category Environment for MFCC feature type.

**Figure A10:**
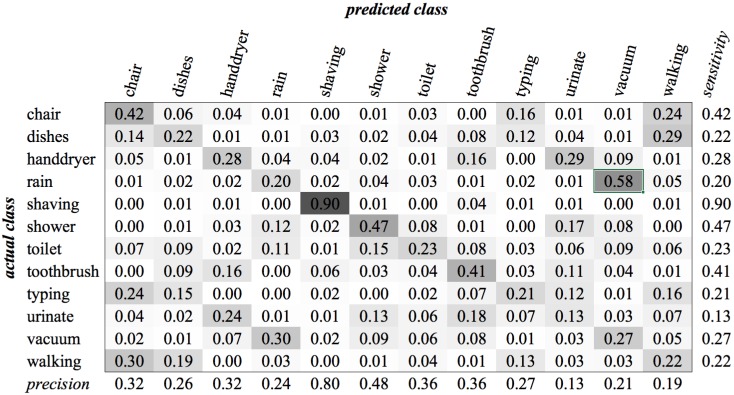
KHMM confusion matrix of category Environment for MFCC feature type.

**Figure A11:**
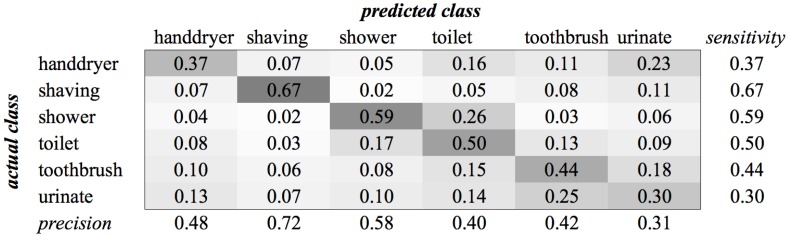
SVM confusion matrix of category Bathroom for MFCC feature type.

**Figure A12:**
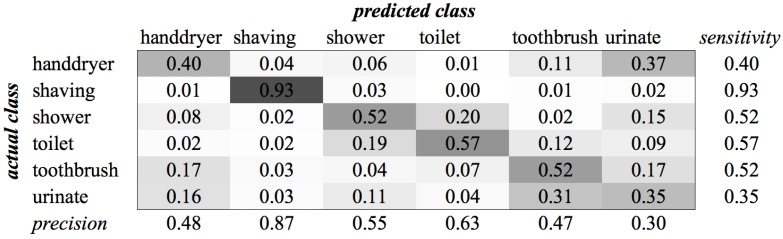
KHMM confusion matrix of category Bathroom for MFCC feature type.

**Figure A13:**
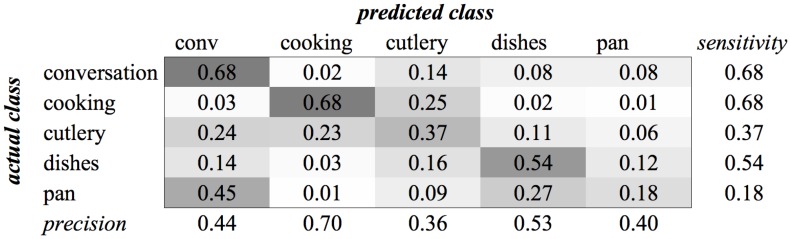
SVM confusion matrix of category Kitchen for MFCC feature type.

**Figure A14:**
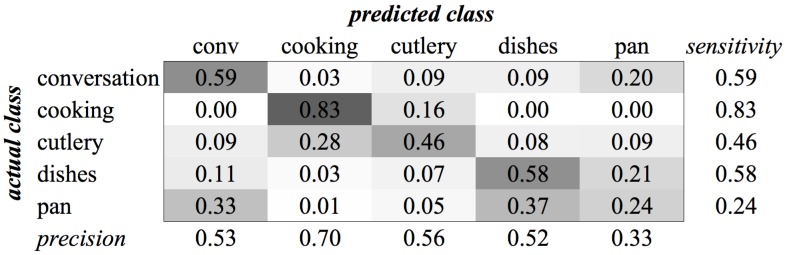
KHMM confusion matrix of category Kitchen for MFCC feature type.

**Figure A15:**
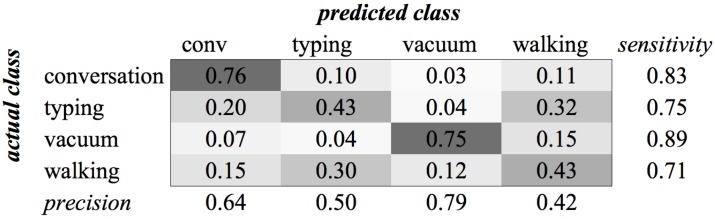
SVM confusion matrix of category Office for MFCC feature type.

**Figure A16:**
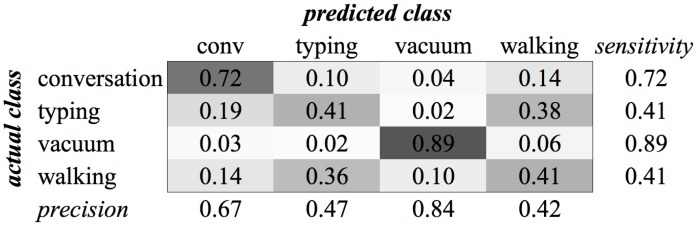
KHMM confusion matrix of category Office for MFCC feature type.

**Figure B1:**
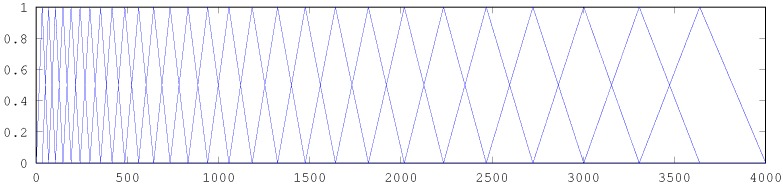
Mel-spaced filterbank.

## Abbreviations

The following abbreviations are used in this manuscript:

FD: Frequency Domain (features)
KHMM: K-means Hidden Markov Model approach
MFCC: Mel-Frequency Cepstral Coefficients
RET: Relative Execution Time
SVM: Support Vector Machine
TD: Time Domain (features)

## Appendix A. Confusion Matrices

In this appendix, we show in more detail the classification results from Table 3 and Table 4. In particular, we show the confusion matrices of the MFCC columns. Note that the numbers in the confusion matrix are normalized for each class. This provides a more balanced overview of the categories that have a high difference in samples per class.

We choose to only show the confusion matrices for the MFCC features, as these features deliver the best classification results in general.

## Appendix B. Feature Algorithm Details

Here we present in more detail how the different features that have been discussed in the previous sections are calculated. In our formulas, we use s(n) to indicate the values of the original sound signal, *N* for the number of sound samples, p(f) to indicate the power spectrum value for a certain frequency, and *F* for the number of Fourier values. We previously described these calculations in [1].

### Appendix B.1. Time Domain

- Zero-Crossing Rate:
  (B1) $$ZCR=\frac{1}{2(N-1)}\sum_{n=1}^{N-1}\left|sgn(s[n])-sgn(s[n-1])\right|$$
  sgn is the signum function.
- Short-Time Energy:
  (B2) $$STE=\sum_{n=0}^{N-1}s{\left(n\right)}^{2}$$
- Sound Amplitude:
  (B3) $$SA=\max\left|s(n)\right|$$
- Peak Detection / Peak Location:
  (B4) $$PL=\underset{n}{\mathrm{argmax}}\left|s(n)\right|$$

### Appendix B.2. Frequency Domain

Frequency analysis depends on the Fourier transform, which is calculated using the Discrete Fourier Transform:

(B5) $$H_{f}\equiv \sum_{n=0}^{N-1}s\left(n\right)e^{-i2\pi f/N}$$

The calculation of the DFT is done using a Fast Fourier Transform algorithm. The power spectrum p(f) is calculated as follows:

(B6) $$p\left(f\right)=\sqrt{Re{\left(H_{f}\right)}^{2}+Im{\left(H_{f}\right)}^{2}}$$

- F0
  (B7) $$F0=\min_{f}\left\{f|p\left(f\right)>\theta \wedge p\left(f-1\right)<p\left(f\right)<p\left(f+1\right)\right\}$$
  where *θ* is a threshold value. Typically, $\theta =0.1\sum_{f}p\left(f\right)$
- The Spectral Centroid for a frame can be defined as:
  (B8) $$SC=\frac{\sum_{f}p(f)*f}{\sum_{f}p\left(f\right)}$$
- The Spectral Rolloff for a frame can be defined as:
  (B9) $$RO=\underset{n}{\mathrm{argmax}}\sum_{f=1}^{n}p\left(f\right)\leq \alpha \cdot \sum_{f=1}^{F}p\left(f\right)$$
  where *α* is a constant with a typical value of 0.97.
- The Bandwidth is defined as:
  (B10) $$BW=\sqrt{\frac{\sum_{f}{\left(f-SC\right)}^{2}*p{\left(f\right)}^{2}}{\sum_{f}p{\left(f\right)}^{2}}}$$
- The Weighted Phase Deviation is defined as:
  (B11) $$WPD=\sum_{f}p\left(f\right)*\phi^{\prime \prime }\left(f\right)$$
  where ϕ(f) is the phase of the Fourier value for frequency *f*.

### Appendix B.3. MFCC

The MFCC are calculated using the following steps:

1. Framing the Signal: the signal is segmented into overlapping frames. The width of the frame is generally about 30 ms with an overlap of about 20 ms.
2. Windowing: A window function is used to smooth the signal for the computation of the DFT so as to minimize the signal discontinuities at the beginning and end of each frame. A typical window is the Hamming window, which has the form
  (B12) $$w\left(n\right)=0.54-0.46\cos\left(\frac{2\pi n}{N-1}\right)$$
  The windowing is calculated using:
  (B13) $$\tilde{s}\left(n\right)=s\left(n\right)w\left(n\right)$$
3. FFT: The power spectrum p(f) of the windowed function $\tilde{s}\left(n\right)$ is calculated as described in Equations (B5) and (B6).
4. The mel filterbanks (see Figure B1) are applied to the power spectrum. The filterbanks are evenly distributed over the frequencies with respect to the Mel scale, which is defined as:
  (B14) $$Mel\left(f\right)=2595\log_{10}\left(1+\frac{f}{700}\right)$$
  The number of filters is 20–40; 26 is standard. This leaves us with 26 numbers that indicate the energy in each filterbank.
5. Take the log of the energies from the previous step.
6. Take the Discrete Cosine Transform (DCT) of the 26 log filterbank energies. This is calculated by the following:
  (B15) $$c_{d}=\frac{1}{M}\sum_{m=0}^{M-1}C_{m}\cos\left(\frac{\pi (2d+1)m}{2M}\right)$$
  where $c_{d}$ is the $d^{th}$ cepstral coefficient, *M* is the total number of filterbanks, and $C_{m}$ denotes the log energy for filterbank *m*. Typically, $c_{1}$–$c_{12}$ constitute the MFCCs.
